# Local CD4 and CD8 T-Cell Reactivity to HSV-1 Antigens Documents Broad Viral Protein Expression and Immune Competence in Latently Infected Human Trigeminal Ganglia

**DOI:** 10.1371/journal.ppat.1003547

**Published:** 2013-08-15

**Authors:** Monique van Velzen, Lichen Jing, Albert D. M. E. Osterhaus, Alessandro Sette, David M. Koelle, Georges M. G. M. Verjans

**Affiliations:** 1 Department of Viroscience, Erasmus MC, Rotterdam, The Netherlands; 2 Department of Medicine, University of Washington, Fred Hutchinson Cancer Research Center, Seattle, Washington, United States of America; 3 La Jolla Institute for Allergy and Immunology, La Jolla, California, United States of America; 4 Department of Laboratory Medicine, University of Washington, Seattle, Washington, United States of America; 5 Department of Global Health, University of Washington, Seattle, Washington, United States of America; 6 Vaccine and Infectious Diseases Division, Fred Hutchinson Cancer Research Center, Seattle, Washington, United States of America; 7 Benaroya Research Institute, Seattle, Washington, United States of America; McMaster University, Canada

## Abstract

Herpes simplex virus type 1 (HSV-1) infection results in lifelong chronic infection of trigeminal ganglion (TG) neurons, also referred to as neuronal HSV-1 latency, with periodic reactivation leading to recrudescent herpetic disease in some persons. HSV-1 proteins are expressed in a temporally coordinated fashion during lytic infection, but their expression pattern during latent infection is largely unknown. Selective retention of HSV-1 reactive T-cells in human TG suggests their role in controlling reactivation by recognizing locally expressed HSV-1 proteins. We characterized the HSV-1 proteins recognized by virus-specific CD4 and CD8 T-cells recovered from human HSV-1–infected TG. T-cell clusters, consisting of both CD4 and CD8 T-cells, surrounded neurons and expressed mRNAs and proteins consistent with *in situ* antigen recognition and antiviral function. HSV-1 proteome-wide scans revealed that intra-TG T-cell responses included both CD4 and CD8 T-cells directed to one to three HSV-1 proteins per person. HSV-1 protein ICP6 was targeted by CD8 T-cells in 4 of 8 HLA-discordant donors. *In situ* tetramer staining demonstrated HSV-1-specific CD8 T-cells juxtaposed to TG neurons. Intra-TG retention of virus-specific CD4 T-cells, validated to the HSV-1 peptide level, implies trafficking of viral proteins from neurons to HLA class II-expressing non-neuronal cells for antigen presentation. The diversity of viral proteins targeted by TG T-cells across all kinetic and functional classes of viral proteins suggests broad HSV-1 protein expression, and viral antigen processing and presentation, in latently infected human TG. Collectively, the human TG represents an immunocompetent environment for both CD4 and CD8 T-cell recognition of HSV-1 proteins expressed during latent infection. HSV-1 proteins recognized by TG-resident T-cells, particularly ICP6 and VP16, are potential HSV-1 vaccine candidates.

## Author Summary

HSV-1 is an endemic human herpesvirus worldwide that establishes a lifelong latent infection of neurons in the trigeminal ganglion (TG), allowing intermittent reactivation resulting in recurrent disease in some persons. Studies in HSV-1 models suggest a central role of TG-infiltrating virus-specific CD8 T-cells to control reactivation. In humans, however, the functional properties and fine specificity of intra-TG T-cell responses remain enigmatic. The current study used molecular, immunological and *in situ* analysis platforms on human cadaveric TG obtained within hours after death to characterize the local HSV-1 specific T-cell response in latently infected human TG in detail. We identified that CD4 and CD8 T-cells were juxtaposed to TG neurons and expressed host transcripts and proteins consistent with *in situ* antigen recognition and antiviral function. The intra-TG T-cell response, involving both CD4 and CD8 T-cells, was directed to a limited set of HSV-1 proteins per person, which was not limited to a specific kinetic or structural class of viral proteins. Collectively, the data indicate that the human TG is an immunocompetent environment for CD4 and CD8 T-cell recognition of diverse HSV-1 proteins expressed during latent infection and that the viral antigens identified herein are rational candidates for HSV-1 subunit vaccines.

## Introduction

The neurotropic human alphaherpesvirus herpes simplex virus type 1 (HSV-1) is endemic worldwide. It is acquired during early childhood via the orofacial route resulting in a lifelong chronic infection of neurons, also referred to as neuronal HSV-1 latency, in the bilateral trigeminal ganglia (TG) [1]. During latency no infectious virus is produced, virus transcription is mainly directed to latency-associated transcripts (LATs) and microRNAs, and HSV-1 proteins are undetectable using standard methods [2]–[7]. Latent HSV-1 periodically reactivates, producing infectious virus that may lead to recrudescent lesions in some persons. Both primary and recurrent disease can result in clinical disorders of variable severity or even death, emphasizing the unmet need for preventive and therapeutic vaccines [1]. The candidate HSV subunit vaccines, based on the HSV glycoproteins B (gB) and gD were tested in human phase III trials, but were not effective [8]–[10]. Vaccines induced antigen-specific antibodies and CD4 T-cells, but not CD8 T-cells, arguing for novel vaccine formulations that include specific HSV-1 antigens targeted by both antibodies as well as CD4 and CD8 T-cells [11].

Studies in humans and HSV-1 mouse models suggest a pivotal role for virus-specific CD8 T-cells in the control HSV-1 reactivation. Virus-specific CD8 T-cells, expressing an activated effector memory T-cell phenotype, are selectively retained in HSV-1–infected ganglia [5], [12]–[15]. In the HSV-1 mouse model with a C57BL/6 background, the HSV-specific intra-TG CD8 T-cells inhibit HSV-1 reactivation by secreting interferon-γ (IFN-γ) and granzyme B (grB), and are mainly directed against an immunodominant HSV-1 gB epitope [16]–[19]. In nature, however, HSV-1 only infects humans. Because HSV-1 infections in mice mimic but are not equivalent to human disease, it is important that findings from mouse models are confirmed and extended to humans [20]–[22]. Moreover, the HSV-1 antigens recognized by human TG-infiltrating T-cells are rational candidates for HSV-1 subunit vaccines.

HSV-1 encodes at least 77 proteins that during lytic infection are sequentially expressed in a coordinated fashion as immediate early (α), early (β), leaky late (γ1) and true late proteins (γ2) [1], [23]. Expression of γ2 proteins depends on viral DNA replication. While infectious virions eventually assemble in distal axonal structures after reactivation, the temporal expression and trafficking of HSV-1 proteins in human neurons during latency is largely unknown. We previously showed reactivity of human TG-derived CD4 and CD8 T-cells to whole HSV-1 [15], but not which proteins were susceptible to local immune recognition. The detection of transcripts encoding the HSV-1 α proteins infecting cell polypeptide 0 (ICP0) and ICP4 in human TG suggests that this kinetic class of proteins is expressed during latency or early after reactivation [5], [7]. However, their accessibility to antigen processing and presentation within TG-resident cells for local T-cell surveillance is unclear. The aims of this study were to characterize the functional properties and HSV-1 antigens recognized by T-cells in HSV-1–infected human TG.

## Results

### Transcripts Levels of T-Cell Cytolytic Effector Molecules and Cytokines Correlate with CD8β Transcript Levels and HSV-1 DNA Load in Human TG

**Figure 1:**
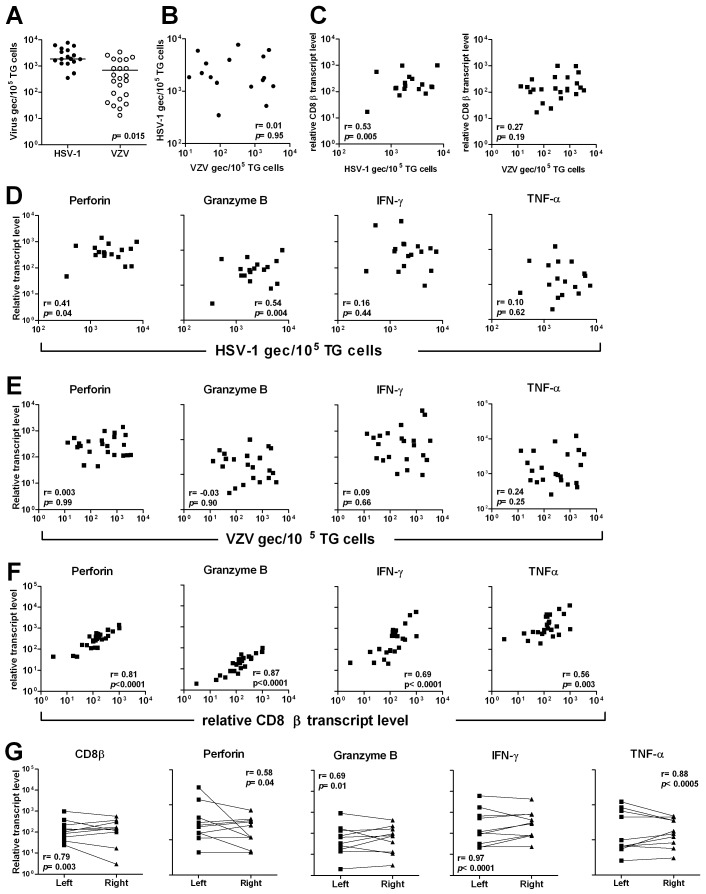
Comparison of T-cell cytolytic granule and cytokine transcripts to HSV-1 and VZV DNA load, and CD8β transcript levels, in human TG. (A) Scatter plot showing the mean HSV-1 and VZV genome equivalent copies (gec) per 10^5^ TG cells. (B) Comparison of HSV-1 and VZV DNA load in TG of individual donors. (C) Comparison between relative CD8β transcript levels and HSV-1 and VZV DNA load. (D) Comparison of HSV-1 DNA load with relative transcript levels of perforin, granzyme B (grB), interferon γ (IFN-γ) and tumor necrosis factor α (TNF-α). (E) Comparison of VZV DNA load with relative transcript levels of perforin, grB, IFN-γ and TNF-α. (F) Comparison between relative CD8β transcript levels and perforin, granzyme B, IFN-γ and TNF-α. (G) Comparison of the transcript levels of CD8β, perforin, grB, IFN-γ and TNF-α between paired left and right TG of individual donors. The paired *T*-test (A), Spearman correlation (B–F) and Wilcoxon matched pairs test (G) were used for statistical analysis. Data of 26 TG specimens analyzed.

In contrast to the HSV-1 mouse models, human TG are commonly co-infected with HSV-1 and the closely related neurotropic human alphaherpesvirus varicella-zoster virus (VZV) [13], [15], [20], [24]. To gain insight into the functional properties of human TG-residing T-cells in relation to latent HSV-1 and VZV we determined the transcript levels of the T-cell cytolytic effector molecules perforin and grB, and the cytokines IFN-γ and tumor necrosis factor-α (TNF-α) in 26 TG of 16 donors by reverse transcriptase real-time PCR [25]. We first determined the prevalence and viral load of latent HSV-1 and VZV by real-time PCR. HSV-1 and VZV DNA was detectable in 17 (65%) and 23 (89%) of the 26 TG analyzed, respectively. Sixteen TG contained DNA of both viruses, seven TG had only VZV, one TG had only HSV-1 and two TG had no detectable DNA of either virus. The presence of virus-specific DNA correlated with the donor's HSV-1 and VZV serostatus and was commonly detected in both TG of each individual (data not shown). Consistent with previous reports, the mean number ± standard error of the mean (SEM) of HSV-1 genome equivalent copies per 10^5^ TG cells (1,850±427) was significantly higher compared to VZV (693±184) (*p* = 0.015) (Figure 1A) [15], [24]. The intra-TG HSV-1 and VZV DNA load did not correlate in paired analysis (Figure 1B). Transcription of the CD8 T-cell–specific gene CD8β correlated weakly with the intra-TG HSV-1 (*p* = 0.005), but not the VZV DNA load (*p* = 0.19) (Figure 1C). Next, the expression levels of the T cell transcripts perforin, grB, IFN-γ and TNF-α were compared to the intra-TG HSV-1 and VZV burden (Fig. 1E and F). A weak correlation was observed between both perforin (*p* = 0.04) and grB (*p* = 0.004) mRNA levels and the intra-TG HSV-1 DNA load, but not with the latent VZV burden (Figure 1D and 1E). Furthermore, the perforin (*p*<0.0001), grB (*p*<0.0001), IFN-γ (*p*<0.0001) and TNF-α mRNA levels (*p* = 0.003) correlated strongly with CD8β mRNA levels (Figure 1F) [5]. The data suggest that the extent of CD8 T-cell infiltration in human TG is not only specifically correlated with the latent HSV-1 burden, but is also transcriptionally active to orchestrate an anti-viral function *in situ*. Finally, the mRNA levels of CD8β (*p* = 0.003), perforin (*p* = 0.04), grB (*p* = 0.01), IFN-γ (*p*<0.0001) and TNF-α (*p*<0.0005) correlated strongly between the paired left and right TG indicating that the intra-TG T-cell responses are symmetric intra-individually (Figure 1G) [15].

### Neuron-Interacting T-Cells in HSV-1 Latently Infected Human TG Express Markers Congruent with T-Cells Recognizing Antigen *In Situ*

**Figure 2:**
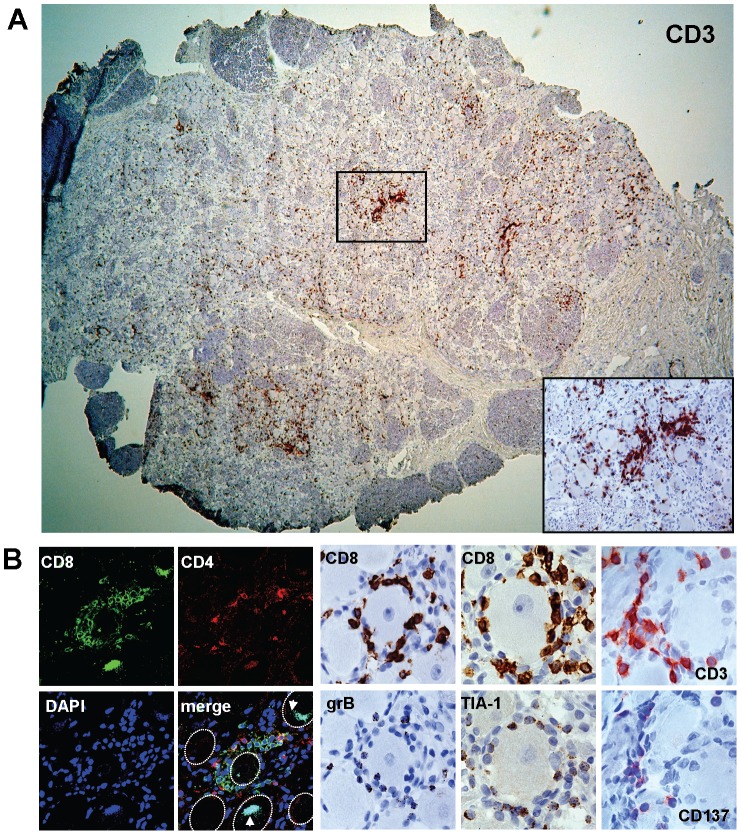
Localization and phenotype of T-cells in HSV-1 latently infected human TG. (A) Representative image of an HSV-1 latently infected TG stained by immunohistochemistry (IHC) for CD3 (red). Inset: magnification of the TG tissue showing a cluster of CD3^+^ cells in panel A. (B; left panel) Double immunofluoresence staining for CD4 (red) and CD8 (green) combined with DNA counterstaining (DAPI; blue nuclei). The white arrows signify autofluorescent cytoplasmatic granules in neurons containing lipofuscin and neuron outlines are marked with white dotted lines. (B; right panel) Consecutive TG tissue sections stained for CD8 (brown) and granzyme B (brown), CD8 (brown) and TIA-1 (brown), and CD3 (red) and CD137 (red). Sections were developed with diaminobenzidine (brown staining pattern) or 3-amino-9-ethylcarbazole (red staining pattern) and counterstained with hematoxylin (blue nuclei). Magnifications were: (A) ×20 and inset ×200, (B; left panel) ×400 and (B; right panel) ×1000. Representative images from 10 HSV-1 latently infected TG donors analyzed.

Compared to HSV-1 negative human TG (Figure S1A), HSV-1 DNA positive human TG are more densely infiltrated with T-cells (Figures S1B), express significantly higher CD8β mRNA levels (Figure S1C) and contain T-cells that on occasion cluster near sensory neuron cell bodies (Figure 2A) [7], [13], [15]. Studies on HSV-1 latently infected sensory ganglia in humans, and particularly in experimentally infected mice, suggest the active role of neuron-interacting T-cells to control neuronal HSV-1 latency [5], [7], [12]–[17]. We first analyzed the presence of TG-infiltrating CD4 and CD8 T-cells by flow cytometry on single cell suspensions of 15 TG of 8 HSV-1 IgG seropositive donors. The data demonstrated infiltration of equivalent numbers of CD4 and CD8 T-cells, with a median ratio of CD4 and CD8 T-cells of 0.99 (range 0.01 to 9.32), which also correlated between the paired left and right TG (*p*<0.0002). Analogous flow cytometric analysis of a TG of one HSV-1 seronegative donor demonstrated a CD4/CD8 T-cell ratio of 1.1, which resembled that of the HSV-1 seropositive donors. However, the limited number of HSV-1 seronegative donors subjected to *ex vivo* flow cytometry analyses withhold conclusions to be drawn on the virus-specific role of retention of either T-cell subtype in human alphaherpesvirus latently infected TG.

Subsequently, we aimed to corroborate the potential protective role of neuron-interacting T-cell clusters by performing detailed *in situ* analyses on HSV-1 latently infected human TG. Neuron-interacting T-cell clusters consisted of both CD4 and CD8 T-cells (Figure 2B). CD8 T-cells expressed both grB and the T-cell intercellular antigen-1 (TIA-1) consistent with their cytotoxic potential (Fig. 2B) [5], [15]. We have recently shown that CD137, a TNF receptor family member [26], is induced on HSV-1 reactive human CD4 and CD8 T-cells shortly after recognition of HSV *in vitro* [27], [28]. Here, we demonstrated that neuron-interacting T-cells in HSV-1 latently infected human TG express CD137 *in situ*, implying that they have encountered their cognate antigen locally (Fig. 2B).

### Human TG-Derived HSV-1-Specific CD8 T-Cells Are Directed to Viral Proteins across All Kinetic and Functional Classes of Viral Proteins

To identify the viral proteins recognized by human TG residing T-cells, T-cell lines (TCL) were generated by mitogenic stimulation of TG-derived T-cells from twelve HSV-1 IgG seropositive donors. The HSV-1–specific T-cells were phenotyped and enumerated by a flow cytometric intra-cellular IFN-γ (IFN-γ ICC) assay using mock– and HSV-1–infected autologous Epstein-Barr virus transformed B-cell lines (BLCL) as antigen-presenting cells (APC). The median percentage of HSV-1–specific CD8 T-cells in the TG-TCL was 10% (range 2 to 37%) and 4 of 12 TG-TCL also contained HSV-1-specific CD4 T-cells (range 0.3 to 10%) (Table 1).

**Table 1:** Phenotype and HLA class I allele restriction of HSV-1 reactive T-cells recovered from human TG.

	TG donor HLA class I genotype			Percentage HSV-1 reactive T-cells			
Donor	HLA-A	HLA-B	ratio	CD4	CD8	HLA-A	HLA-B
ID	allele 1; 2	allele 1; 2	CD4/CD8#	T-cells*	T-cells*	allele 1;2$	allele 1;2$
TG1	A*0201; A*1101	B*0702; B*4402	0.7	nd	5	4; 0	1; 0
TG2	A*0201	B*1501; B*4402	0.8	5	18	5	13; 0
TG3	A*0101	B*0801	5.4	0.3	11	11	0
TG4	A*0201; A*0301	B*3501; B*4402	nd	0	2	2; 0	0; 0
TG5	A*0301; A*3004	B*3501; B*4001	0.2	0	7	0; nd	0; 7
TG6	A*0301; A*2902	B*0702; B*4403	0.2	10	22	2; 1	19; 0
TG7	A*0301; A*3101	B*4001; B*5101	0.5	0	37	10; 9	18; 0
TG8	A*0101; A*02	B*07; B*0801	21.2	0	10	2; 3	5; nd
TG9	A*0201; A*6802	B*1402; B*5701	0.8	nd	5	1; 1	3; 0
TG10	A*0201; A*0301	B*3501	14.7	0	24	2; 22	0
TG11	A*0101; A*2902	B*0801; B*4403	2.9	nd	11.5	3; 2	3; 3.5
TG12	A*1101; A*3101	B*4001	0.7	4	10	3; 0	7

* TG-derived T-cell lines were incubated with mock– and HSV-1–infected autologous B-cell lines and assayed by flow cytometry for intra-cellular interferon gamma (IFN-γ) expression.

# The ratio of CD4 and CD8 T-cells of the respective TG-derived T-cell lines are indicated.

$ Patient HLA class I allele restricted HSV-1 reactive CD8 T-cell responses were defined using partially HLA class I matched BLCL. The values represent mean net percentages of live/CD3-gated IFN-γ^+^ T-cells (HSV-1 minus mock) of at least 2 separate experiments.

nd, not done.

The HSV-1 proteins recognized by human TG-derived CD8 T-cells were determined using transfected Cos-7 cells as artificial APC that expressed one of the donor's HLA-A and –B alleles in combination with 74 separate HSV-1 open reading frames (ORFs) [27]. First, we used a set of partially HLA–A or –B allele matched HSV-1-infected BLCL as APC to uncover both the diversity and identity of HSV-1 peptide-presenting HLA class I (HLA-I) alleles used by the CD8 T-cells. The data demonstrated that the virus-specific intra-TG CD8 T-cell response is mediated by 1 to 4 different HLA–A and –B alleles per person (Table 1). Next, we used the implicated HLA-A and -B allele in HSV-1 ORFeome-wide screen [27]. We observed definitive HSV-1 ORFeome screen hits only when the net proportion of CD8 T-cells reactive with HLA-matched HSV-1-infected BLCL was >4% (data not shown). For some donors, we enriched and expanded the TG-TCL using CD137 selection [27], [28]. For this, TG-TCL were incubated with HSV-1–infected autologous BLCL and CD137^+^ CD8 T-cells were selected after 18 hours of incubation and expanded with a T-cell mitogen to generate a second generation TG-TCL. This led to an approximately 3-fold enrichment of HSV-1 reactive CD8 T-cells for donors TG1, TG4 and TG12. The TG-TCL of donors TG8, TG9 and TG11 yielded insufficient enrichment or ample T-cell numbers to perform HSV-1 ORFeome screens (data not shown).

**Figure 3:**
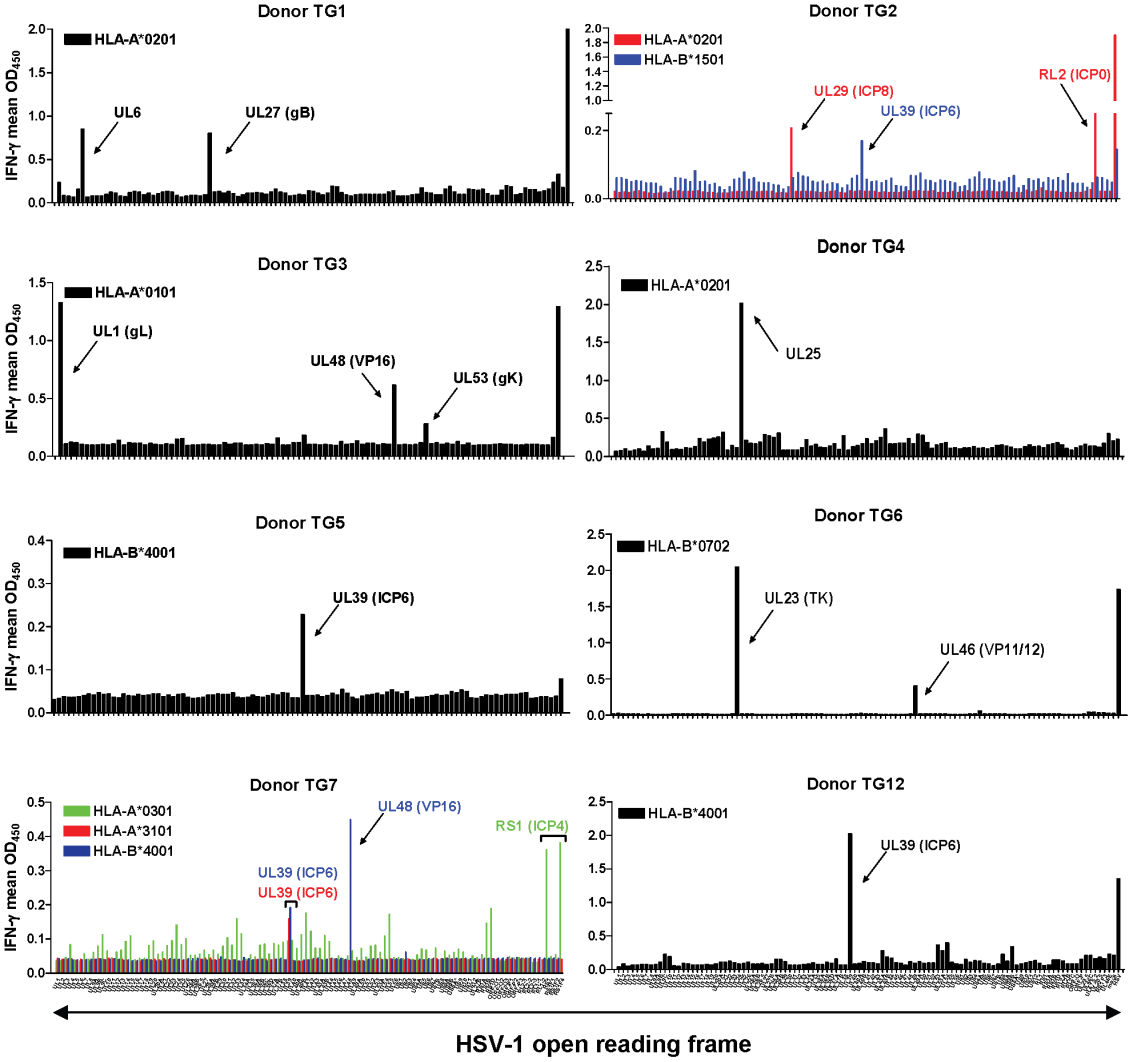
HSV-1 antigens recognized by human TG-derived CD8 T-cells. Representative data from TG-TCL of 6 TG donors assayed for T-cell reactivity to proteins encoded by individual HSV-1 open reading frames (ORFs). Mean IFN-γ secretion levels, shown as arbitrary OD_450_ values, by TG-TCL exposed in duplicate to Cos-7 cells that co-express the respective donor-specific HLA class I allele and the individual HSV-1 ORFs arrayed in nominal genomic order on the x-axis. The names of the HSV-1 ORFs and corresponding proteins specifically recognized are indicated by an arrow. The HSV-1 ORFs recognized by the TG-TCL of donors TG2 and TG7 are shown in colors of the respective TG donor-specific HLA class I allele. ICP, infected cell polypeptide; VP, virion protein and in case of a viral glycoprotein (e.g. gB, glycoprotein B). Currently, no proteins names are available for HSV-1 ORFs *UL6* and *UL25*.

In total, 8 of 12 HSV-1 reactive TG-TCL revealed reproducible specific HSV-1 CD8 T-cell antigen hits (Figure 3). Thirteen different CD8 T-cell viral targets were identified with 1 to 3 viral proteins per TG-TCL (Figure 3). Virion protein 16 (VP16) and particularly ICP6 were recognized by multiple TG-TCL in the context of diverse HLA-A and –B alleles. In case of ICP6, 4 of 8 TG-TCL were positive and the protein was recognized via HLA-A*3101 (donor TG7), –B*1501 (donor TG2) and in 3 different TG donors via HLA-B*4001 (donors TG5, TG7 and TG12) (Figure 3). Finally, candidate CD8 T-cell epitopes within several HSV-1 screen hit proteins were predicted by *in silico* algorithms [29]. Epitopes were subsequently validated by IFN-γ ICC using corresponding synthetic peptides and HLA-matched BLCL as APC (Figures S2 and S3 and Table 2).

**Table 2:** HSV-1 antigens and epitopes recognized by CD8 T-cells recovered from human TG.

		Kinetic class of the HSV-1 proteins recognized*		
Donor ID	HLA allele#	Immediate early	Early	Late
TG1	A*0201	na	na	UL6 protein
TG1	A*0201	na	na	gB
TG2	A*0201	ICP0 (aa 642–651)	ICP8 (aa1096–1105)	na
TG2	B*1501	na	ICP6	na
TG3	A*0101	na	na	gL (aa 66–74)
TG3	A*0101	na	na	gK (aa 201–209)
TG3	A*0101	na	na	VP16 (aa 90–99)
TG3	A*0101	na	na	VP16 (aa 479–488)
TG4	A*0201	na	na	UL25 protein
TG5	B*4001	na	ICP6	na
TG6	A*2902	na	na	VP13/14 (aa 508–516)
TG6	B*0702	na	Thymidine kinase	VP11/12 (aa 386–394)
TG7	A*0301	ICP4 (aa 1096–1105)	na	na
TG7	A*3101	na	ICP6	na
TG7	B*4001	na	ICP6	VP16 (aa 163–175)
TG12	B*4001	na	ICP6	na

* HSV-1 gene and protein names, and expression kinetic class, are from reference 1 and Genbank NC_001806. The amino acid (aa) location of CD8 T-cell epitopes identified are in parentheses. na, not applicable.

# HLA allele by which the indicated proteins and peptides are recognized by the specific CD8 T-cells.

We recently studied antigenic targets of blood-derived HSV-1 specific CD8 T-cells in HSV-1 IgG seropositive healthy subjects using related methodologies [27]. Systemic HLA–A and –B restricted CD8 T-cell responses were directed to 14 HSV-1 ORFs on average per person and 45 HLA–A and –B allele restricted HSV-1 epitopes were identified [27]. To discern potential similarities between systemic and intra-TG HSV-1 peptide specific CD8 T-cell responses we tested TG-TCL of seven HLA-A and -B allele matched TG donors for responses to the HLA-appropriate HSV-1 peptides from our previous work on blood-derived T-cells [27] (Table S1). Peptide-specific CD8 T-cell responses were detected in two TG-TCL. The TG-TCL of donor TG3 recognized four HLA-A*0101–restricted peptides: gL_66–74_, gK_201–209_, and two VP16 peptides VP16_90–99_ and VP16_479–488_. The HLA-A*2902–restricted VP13/14_508–516_ peptide was recognized by the TG-TCL of donor TG6 (Table 2 and Table S1) [27].

Collectively, the data demonstrated that human intra-TG HSV-1–specific CD8 T-cell responses were directed to a relatively restricted number of viral proteins per person. However, even within the small population studied, we detected CD8 T-cell responses to HSV-1 proteins in diverse kinetic and structural classes (Table S2). Notably, the HSV-1 β protein ICP6 was a prominent CD8 T-cell target in TG-TCL of 4 of 8 HLA-discordant TG donors involving 3 different HLA-I alleles.

### HSV-1-Specific Human TG-Derived CD4 T-Cells Recognize Immediate Early and Late Viral Proteins

**Figure 4:**
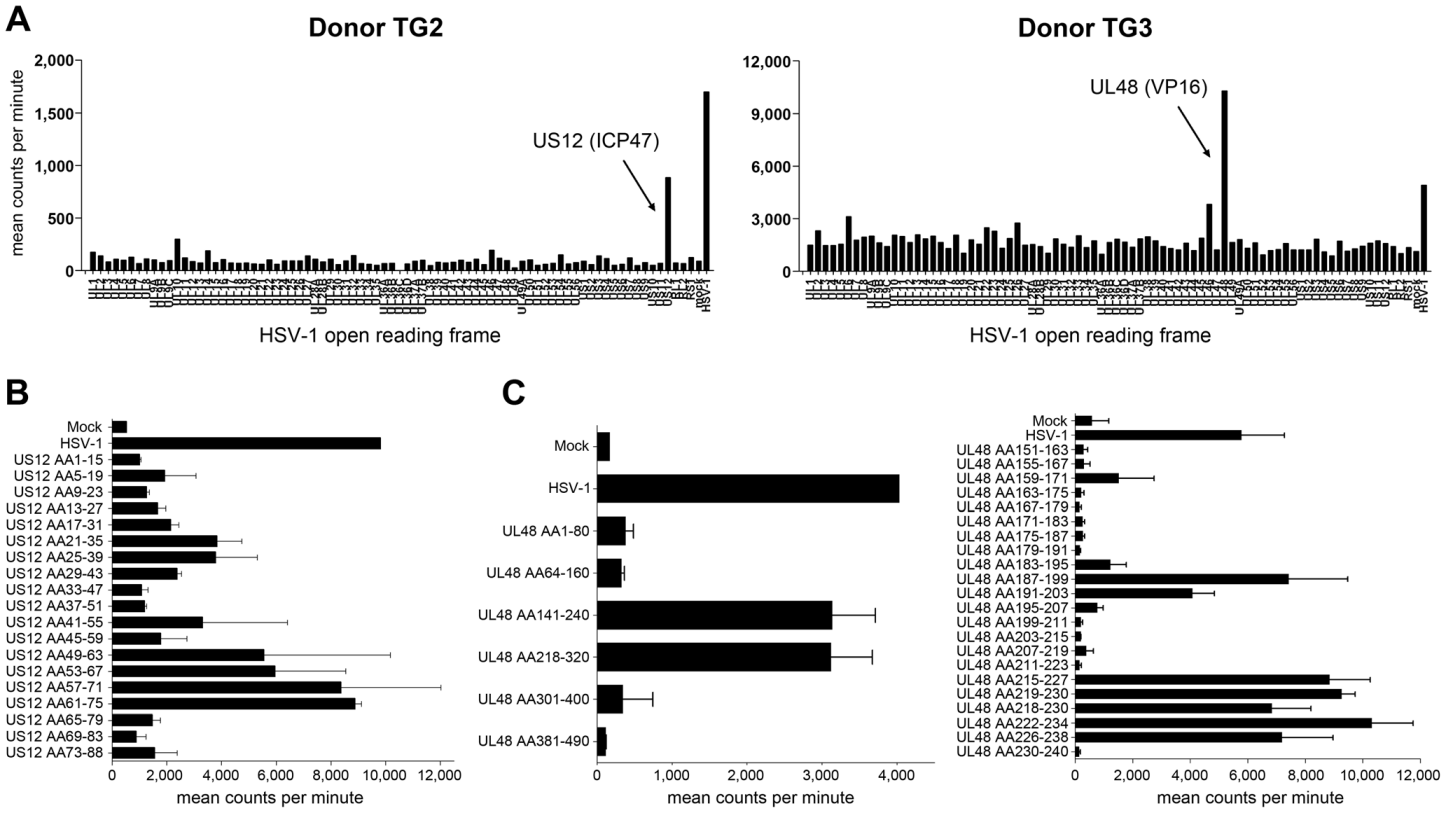
HSV-1 antigens recognized by human TG-derived CD4 T-cells. (A) Representative data from the TG-TCL of the donors TG2 and TG3 assayed for T-cell reactivity by a proliferation assay to proteins encoded by individual HSV-1 open reading frames (ORFs). Cell lysates of mock- and HSV-1 infected Cos-7 cells were used as negative and positive controls, respectively. Mean [^3^H]-thymidine incorporation by TG-TCL exposed in duplicate to γ-irradiated donor HLA-DQ/DR-matched allogeneic peripheral blood mononuclear cells (PBMC) pulsed with lysates generated from Cos-7 cells transfected with the individual HSV-1 ORFs arrayed in nominal genomic order on the x-axis. The names of the HSV-1 ORFs and corresponding proteins driving the positive responses are indicated by an arrow. ICP47, infected cell polypeptide 47 and VP16, virus protein 16. (B) Proliferation assay data of the TG-TCL of donor TG2 with γ-irradiated HLA-DQ/DR TG 2-matched allogeneic PBMC pulsed with whole HSV-1 ICP47 protein (gene US12) spanning synthetic peptides (15-meric peptides with 10 amino acid (aa) overlap) as antigen presenting cell (APC). (C; left panel) Proliferation assay data of the TG-TCL of donor TG3 with γ-irradiated HLA-DQ/DR-matched allogeneic PBMC pulsed with the indicated recombinant HSV-1 VP16 protein (gene UL48) fragments as APC. (C; right panel) Proliferation assay data of the TG-TCL of donor TG3 with γ-irradiated HLA-DQ/DR-matched allogeneic PBMC pulsed with HSV-1 VP16 protein fragment (aa151–240) spanning synthetic peptides (13-meric peptides with 8 aa overlap) as APC. Data are presented as mean counts per minute of triplicate experiments. Data presented in (B) and (C) are the means ± standard error of the mean.

The intra-TG CD4 T-cell responses were analyzed in detail for donors TG2 and TG3 (Table 1) [28]. CD4 T-cells of donor TG2 responded to the HSV-1 α protein ICP47 and subsequent assays using whole ICP47-spanning peptides defined the antigenic region at residues 57–75 (Figure 4A and 4B). For donor TG3, CD4 T-cell reactivity was directed to the HSV-1 γ1 protein VP16 (Figure 4A). Application of truncated recombinant VP16 proteins and subsequently overlapping peptides identified two distinct antigenic regions located between residues 187–203 and 215–238 (Figure 4C). Besides being a structural viral protein, VP16 has also been implicated as a master initiator protein for HSV-1 neuronal reactivation in mice [30].

### HSV-1 Epitope Specific CD8 T-Cells Localize in Close Proximity to Sensory Neuron Cell Bodies in Human TG

**Figure 5:**
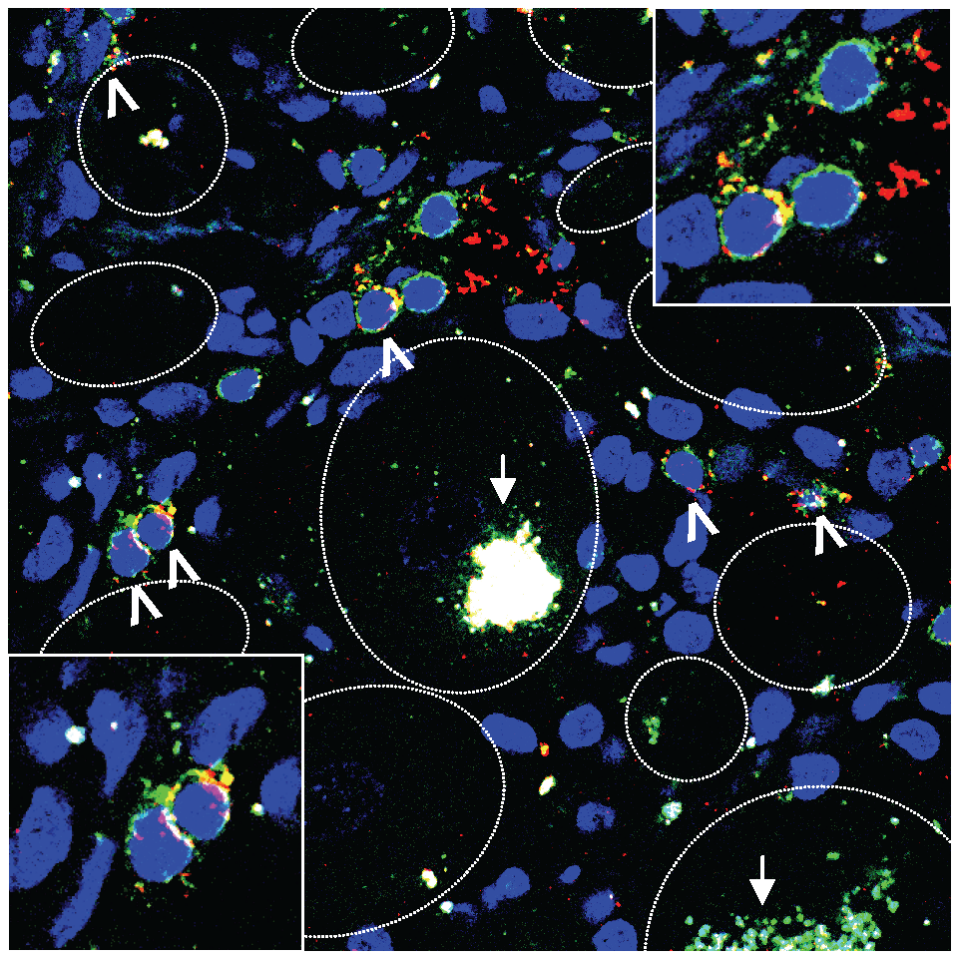
HSV-1 epitope-specific CD8 T-cells are localized close to sensory neuron cell bodies in the contralateral human TG. Representative image of the TG tissue of donor TG2 stained with DAPI (blue), anti-CD8 (green) and tetramers (red) that consisted of both the synthetic HSV-1 peptides ICP0_642–651_ and ICP8_1096–1105_ conjugated to HLA-A*0201. Inserts, lower left and upper right corner, are enlargements of areas containing tetramer-positive CD8 T-cells. The white arrows and arrowheads signify autofluorescent granules containing lipofuscin and tetramer-positive CD8 T-cells, respectively. Neuron outlines are marked with a white dashed line. Magnification was ×400.

The symmetry of the virus and T-cell parameters between paired TG (Figure 1) [15], facilitated studies on the spatial orientation of HSV-1 reactive CD8 T-cells in the contralateral snap-frozen TG specimen of the same donor by *in situ* tetramer staining (Figure 5) [31]. HSV-1 CD8 T-cell epitopes and corresponding snap-frozen contralateral TG specimens were available for donors TG2 and TG3. HLA-A*0201 tetramers conjugated with identified ICP0_642–651_ and ICP8_1096–1105_ epitopes and HLA-A*0101 reagents with gL_66–74_, gK_201–209_, VP16_90–99_ and VP16_479–488_ epitopes (Table 2), were validated on the corresponding TG-TCL (Figures S2 and S3). Whereas control TG of HLA-A mismatched HSV-1 seropositive donors did not reveal tetramer-positive CD8 T-cells (data not shown), HSV-1 tetramer-positive CD8 T-cells were found juxtaposed to neuronal cell bodies in TG of the respective donor (Figures 5 and S4, and Movie S1).

## Discussion

The host-pathogen standoff in human latent HSV-1 infection permits periodic epithelial shedding of infectious virus, and potential transmission, without overt host damage. In contrast, HSV-1 mouse models are either acutely fatal or demonstrate tight neuronal latency in which neither spontaneous nor stress-induced ganglionic reactivation leads to peripheral release of infectious virus [17], [20], [32]. In the current study, we demonstrated that the human TG is an immunocompetent organ capable of presenting viral antigens to both CD4 and CD8 T-cells, presumably over long periods of time, to maintain local enrichment of HSV-1–specific T-cells. The data imply that the HSV-1 proteins expressed in latently infected human TG are not limited to a specific class of kinetic or structural viral proteins and that the viral antigens identified herein are rational candidates for HSV-1 subunit vaccines.

In contrast to viral DNA and transcripts, viral proteins have not been detected in HSV-1 latently infected human ganglia [5], [7], [13]. Viral protein synthesis may be shutdown or occur at low levels or at low frequency. T-cells are activated by only a few MHC/peptide-complexes making them highly sensitive and specific biosensors to detect extremely low-level expression of their cognate antigens [33]. The recognition of diverse HSV-1 proteins by human TG infiltrating T-cells implies their cognate antigen expression *in situ*. Moreover, the HSV-1 targets identified did not group to a specific kinetic or functional class of viral proteins suggesting that HSV-1 protein synthesis is not limited to early viral proteins in latently infected human TG [5], [7]. Alternatively, the T-cell response reported herein may be directed to local reactivating HSV-1, which has overcome cellular and viral control including HSV-1 micro RNAs [6], [7]. In contrast to humans, HSV-1 mouse models are either fatal or have tight neuronal latency in which spontaneous reactivation does not lead to peripheral release of infectious virus [20], [21]. Nevertheless, latently infected TG of C57BL/6 mice contain neuron-interacting CD8 T-cells, directed to 11 viral proteins including late structural HSV-1 proteins like gC and gK [12], [18], [19]. These data imply that full HSV-1 reactivation is not a prerequisite to retain virus-specific T-cells in ganglia with diverse viral protein reactivity. The combined human and mouse data argue that this process involves recognition of the T-cells' cognate viral antigens produced locally in HSV-1 latently infected ganglia [5], [15], [18], [20].

Activation of HSV-1-specific CD8 T-cells in latently infected murine ganglia is dependent on local CD4 T-cells, MHC class II expression and recruited blood-derived APC [14], [34]. The current study documents inclusion of CD4 T-cells in neuron-interacting T-cell clusters and proves peptide-level recognition of HSV-1 by TG-resident CD4 T-cells in the natural host. Intriguingly, VP16 was targeted by both CD8 and CD4 T-cells in the same TG specimen (i.e., TG3) indicating local expression and presentation of this viral protein by TG cells in the context of both HLA-I and HLA-II molecules. Although local human APC driving the HSV-1-specific CD4 T-cell responses could be blood-derived or ganglion-resident [14], [34], it is unlikely that HLA class II negative neurons are directly involved. The data suggest of HSV-1 proteins or remnants thereof from neurons to secondary APC. We have recently shown that satellite glial cells (SGC), which tightly envelop neuronal cell bodies in ganglia, are most likely of myeloid origin [35]. Human TG-resident SGC are related to macrophages and myeloid dendritic cells with regards to their phagocytic capacity and expression of CD45, co-stimulatory and HLA class II molecules [23]. Given their localization and phenotype, SGC are candidate APC to create an immunocompetent but not overtly inflammatory environment to support HSV-specific CD4 and CD8 T-cell responses within latently infected human TG.

An important and still unanswered question is the functional role of the HSV-specific T-cells documented in this report. In the absence of tools to selectively interrupt or bolster T-cells at specific anatomic sites in humans, this question is difficult to address. Surrogate data can be obtained by examining the phenotype and activation status of human TG-resident CD8 T-cells. Integrating human TG *ex vivo* flow cytometry and *in situ* data, it is evident that TG-resident CD8 T-cells express CD137 and grB, and low levels of CD27 and CD28, indicative of recent antigen encounter locally [5], [12], [14], [15], [36]. Messenger RNA expression of the T-cell cytolytic molecules perforin and grB directly correlated with HSV-1 DNA levels (Figure 1D) and grB and TIA-1 protein expression co-localized with neuron-surrounding CD8 T-cells (Figure 2B). The fact that human TG-resident HSV-1-specific CD8 T-cells can massively expand *in vitro* and then display brisk virus-specific IFN-γ responses argues against an exhausted phenotype [15], [37]. Taken together with the remarkable localization of HSV-1-specific CD8 T-cells juxtaposed to TG neurons (Figures 5 and S4 and Movie S1), our data argue for a functional role for these cells in non-lytic control of HSV-1 infection in human TG in cooperation with local CD4 T-cells.

If this interpretation is correct, elicitation of T-cells with anti-viral activity in the latently infected TG is a rational goal for preventative and therapeutic HSV-1 vaccines. Our findings have several implications for HSV-1 vaccine design. First, ICP6 was recognized by 4 of 8 TG donors in diverse HLA-I contexts (Table 2). ICP6 is a ribonucleotide reductase subunit expressed prior to viral DNA replication [1]. Because ICP6 was also a dominant target for the systemic CD8 T-cell response in HSV-1 seropositive subjects [27], this protein is an attractive vaccine candidate. Second, HSV-1 proteins of diverse kinetic and structural classes were recognized by TG CD8 T-cells (Table 2). These range from nonstructural α (ICP0 and ICP4) and β proteins (ICP6 and thymidine kinase) to the late structural tegument (VP11/12 and VP13/14) and envelope glycoproteins (gB, gK and gL). Tegument protein VP16, recognized by both CD4 and CD8 TG-resident T-cells, is possibly a chameleon with both a hyper-early role in neuronal reactivation and a structural role in tegument assembly [30]. The cell biology implication of this finding appears that diverse HSV-1 proteins are diverted from viral assembly and access the HLA class I pathway in neurons, or possibly surrounding APC after handover. Third, the apparent diversity of recognized HSV-1 antigens is lower in TG than in blood, where we detected a mean of 14 reactive HSV-1 ORFs per person using similar technology [27]. The restricted clonality of the human intra-TG T-cell response is consistent with T-cell receptor spectratyping [5], [15], [36]. Surveys of more participants, ideally with parallel studies on blood-derived T-cells, are mandatory to determine if the breadth or fine specificity of the paired TG and systemic HSV-1 T-cell responses differ and to pick the best antigens for possible subunit approaches that target sensory ganglia as a site of viral control. Manipulation of T-cell priming or boosting to imprint a TG-homing program via vaccination, without imparting an overly aggressive phenotype, is an equally important and challenging task that must be overcome to target the TG as an immunocompetent site for the purpose of HSV-1 latency control.

## Materials and Methods

### Ethics Statement

Heparinized peripheral blood and paired TG specimens were obtained from 39 individuals (median age 71 yrs, range 49–98 yrs) at autopsy with a median post-mortem interval of 6.3 hrs (range 2.3–11.3 hrs). Specimens were collected by the Netherlands Brain Bank (Netherlands Institute for Neuroscience; Amsterdam, the Netherlands) from donors from whom a written informed consent for brain autopsy and the use of the material and clinical information for research purposes had been obtained. All study procedures were performed in compliance with relevant Dutch laws and institutional guidelines, approved by the local ethical committee (VU University Medical Center; Amsterdam, project number 2009/148) and was performed in accordance with the ethical standards of the Declaration of Helsinki. The majority of the TG donors (n = 31) had a neurologic disease history affecting the central nervous system (mainly Alzheimer's and Parkinson's disease). Causes of death were not related to herpesvirus infections. Blood was used to generate BLCL and for HLA typing as described [15], [27]. Plasma HSV-1 and VZV IgG levels were determined by ELISA (Focus Diagnostics).

### Generation and HSV-1-Specificity Testing of TG-Derived TCL

TG-TCL were generated by phytohemagglutinin (PHA) stimulation of TG cell suspensions, or of CD137-enriched TG-TCL, using γ-irradiated allogeneic PBMC and recombinant human IL-2 as described [15]. Antigen-specificity of TG-TCL was determined by IFN-γ ICC using the following APC: autologous or partially HLA class I-matched BLCL infected overnight with HSV-1 with a multiplicity of infection (MOI) of 10, or BLCL pulsed with 2 µM of HSV-1 peptides [27]. Mock-infected BLCL were used as negative controls. Cells were stained for CD4, CD8, CD3 and IFN-γ (all from Becton Dickinson; BD) and analyzed by multicolor flow cytometry with Diva software (BD) as described [15]. *Ex vivo* flow cytometry analyses for CD3, CD4, CD8 expression was performed on single TG cell suspensions of a subset of 16 TG specimens as described [15].

### Isolation of Nucleic Acids and PCR Analysis of Human TG Specimens

One-fifth of a dispersed TG cell suspension was used for RNA and DNA isolation [15]. RNA was reverse transcribed using an oligo-dT primer and used for quantitative real-time PCR (qPCR) on an ABI Prism 7700 with Taqman Universal Master Mix and commercial intron-spanning primer/probe-pairs specific for human perforin, grB, CD8β, TNF-α, IFN-γ and β-actin (Applied Biosystems) per manufacturer. The relative transcript levels were determined by the formula 1,000×2^(−delCt)^, where delCt equals Ct [(target gene) - Ct (β-actin)]. Intra-TG HSV-1 and VZV DNA load were determined by qPCR as described [25].

### CD137-Based Enrichment of Virus-Specific CD8 T-Cells from TG-Derived TCL

To enrich HSV-1 reactive CD8 T-cells, autologous BLCL were infected overnight with HSV-1 with a MOI of 10. TG-TCL were added to the APC at a ratio of 1∶1 for the next 24 hrs. Cells were harvested, stained for CD3 (BD), CD8 (BD) and CD137 (Miltenyi). Cells that co-expressed CD3, CD8, and CD137 were enriched with a BD FACS Aria cell sorter, expanded by PHA stimulation and used in HSV-1 ORFeome screens as described [27], [28].

### T-Cell Reactivity to HSV-1 ORFeome

The generation and validation of the HSV-1 ORFeome, covering a total of 74 HSV-1 ORFs, for functional T-cell assays has been detailed elsewhere [27]. In short, each HSV-1 ORF was amplified and cloned into a custom-made eukaryotic expression vector fused to the gene encoding enhanced green fluorescent protein (eGFP). Donor-matched HLA-I specific cDNA (in pcDNA3) and HSV-1 ORFs were expressed in Cos-7 cells (ATCC CRL-1651) by transfection [27]. All HSV-1 ORFs were transfected in duplicate and appropriate mock- or HSV-1 infection controls were included. After 48 hrs, ORF expression was confirmed by eGFP fluorescence and TG-TCL (5×10^4^/well) were added to 10^4^ transfected Cos-7 cells/well. After 24 h, supernatants were collected for IFN-γ ELISA [27].

Whole HSV-1 ORFeome screens to identify CD4 T-cell target antigens were performed in duplicate as described [28], [38]. Gamma-irradiated HLA-DQ/DR-matched allogeneic PBMC were pulsed overnight with predefined dilutions of protein lysates of HSV-1 ORF-transfected Cos-7 cells, HSV-1 proteins made with bacterial lysates or peptides at 2 µM [28], [38]. Ultraviolet light treated mock- and HSV-1-infected Vero cell lysates were used as negative and positive controls, respectively. After 48 hrs, [^3^H]-thymidine was added and cells harvested to measure [^3^H]-thymidine incorporation as marker for T-cell proliferation [27], [38].

### *In Situ* Analyses of Human TG Specimens

*In situ* immunofluorescence was performed using allophycocyanin (APC)-labeled CD4 (clone RPA-T4; BD) and FITC-labeled CD8 (1A5; Monosan) monocloncal antibodies (mAbs). The APC signal was enhanced by the FASER system per manufacturer (Miltenyi). Sections were post-fixed with 4% (w/v) formaldehyde, counterstained for DNA with DAPI (Invitrogen) and mounted with ProLong Gold Antifade Reagent (Invitrogen). For immunohistochemistry, paraffin sections and cryosections of human TG were stained as described [35]. The mAbs used were directed to CD8 (1A5; Monosan), grB (GrB-7; Dako), TIA-1 (2G9; Immunotech), CD3 (UCHT1; Dako) and CD137 (4B4-1; BD). Sections were counterstained with hematoxylin and mounted with glycerol gelatin. The ratio of CD3^+^ cells per neuron in TG of HSV-1 serotyped donors was determined by counting all sensory neuronal cell bodies and CD3^+^ cells in multiple sections (n = 3–5), cut at different anatomic levels of the same TG specimen, under the microscope as described previously [39]. The average number CD3^+^ cells/neuron per TG are presented.

*In situ* tetramer stainings were performed as described previously [31]. In brief, TG cryosections (8 µm) were fixed with 4% (w/v) formaldehyde and incubated with 2–4 µg of the respective APC-conjugated HSV-1 peptide/HLA-I tetramers at 4°C for 20 hrs. Next, slides were washed and post-fixed in 4% formaldehyde. Slides were counterstained with anti-CD8 (3B5; Invitrogen) and DAPI (Invitrogen), and mounted with ProLong Gold Antifade Reagent (Invitrogen). Fluorescent images were acquired on a Zeiss LSM700 confocal laser scanning microscope.

### Statistical Analysis

Statistical differences between were determined by the Mann-Whitney test, paired *T*-test, Spearman correlation test and Wilcoxon matched-pairs signed-rank test. *P*<0.05 were considered significant.

## Supporting Information

Figure S1 **T-cell retention in human TG correlates with HSV-1 latency.** (A) Representative image of a HSV-1 negative human TG stained by immunohisto-chemistry for CD3 (red). Sections were developed with 3-amino-9-ethylcarbazole (red staining pattern) and counterstained with hematoxylin (blue nuclei). Magnification was ×20. Note the limited number of infiltrating CD3^+^ cells that, in contrast to a TG of a representative HSV-1 positive donor (see Fig. 2A), did not form neuron-interacting T-cell clusters. Representative image from 5 HSV-1 negative TG donors analyzed. (B) Scatter plot showing the mean number of CD3^+^ cells per neuronal cell body in TG of HSV-1 seropositive (n = 7) and seronegative (n = 3) individuals. (C) Scatter plot showing the mean relative CD8β transcript levels in human TG that are HSV-1 DNA positive (n = 17) or negative (n = 9). (B and C) The Mann-Whitney test was used for statistical analysis.
(TIFF)

Figure S2 **Validation of HSV-1 peptide-specific tetramers on the TG-TCL of donor TG2.** The TG-TCL of donor TG2 were incubated with mock- and -HSV-1 infected, and peptide-pulsed, autologous B-cell lines for 16 hrs. Gated live and CD3^+^ cells were assayed by flow cytometry for intra-cellular gamma interferon (IFN-γ), and surface CD3 and CD8 expression. Additionally, the donor's TG-TCL, or a relevant HLA-A allele mismatched TG-TCL, were incubated with the corresponding fluorochrome-conjugated HLA class I tetramers for 1 hr and binding determined on live gated cells in combination with CD3 and CD8 staining (lower row). Numbers are percentages of cells in the upper right quadrant. aa, amino acid; ICP, infected cell polypeptide.
(TIF)

Figure S3 **Validation of HSV-1 peptide-specific tetramers on the TG-TCL of donor TG3.** The TG-TCL of donor TG3 were incubated with mock- and -HSV-1 infected, and peptide-pulsed, autologous B-cell lines for 16 hrs. Gated live and CD3^+^ cells assayed by flow cytometry for intra-cellular gamma interferon (IFN-γ), and surface CD3 and CD8 expression. Additionally, the donor's TG-TCL, or a HLA-A allele mismatched TG-TCL, were incubated with the corresponding fluorochrome-conjugated HLA class I tetramers for 1 hr and binding determined on live gated cells in combination with CD3 and CD8 staining (lower row). Numbers are percentages of cells in the upper right quadrant. aa, amino acid; VP, virus protein; gK, glycoprotein K and gL, glycoprotein L.
(TIFF)

Figure S4 **HSV-1 epitope-specific CD8 T-cells are localized in the vicinity to sensory neuron cell bodies in human TG tissue.** Representative optical sections from snap-frozen TG tissue of donor TG3 stained with DAPI (blue), anti-CD8 (green) and tetramers (red) that consisted of the synthetic HSV-1 peptides gL_66–74_ (upper panel), VP16_90–99_ (middle panel) and gK_201–209_ (lower panel) bound to HLA-A*0101. The white arrows and arrow heads signify autofluorescent granules containing lipofuscin and tetramer-positive cells, respectively. Boxed areas in the upper and middle panels are enlarged in the corresponding images to the right. Note that for the HLA-A*0201/VP16_90–99_ tetramer staining anti-CD8 was omitted. Neuron outlines are marked with a white dashed line. Magnifications were ×400 and in the insets ×800.
(TIFF)

Movie S1 Three-dimensional reconstruction of optically sectioned snap-frozen TG tissue of donor TG2 stained with DAPI (blue), anti-CD8 (green) and tetramers (red) that consisted of both the synthetic HSV-1 peptides ICP0_642–651_ and ICP8_1096–1105_ conjugated to HLA-A*0201. Image stack size is 75 (x)×75 (y)×8 (z) µm.
(MOV)

Table S1 **HSV-1 peptide responses in TG-TCL of HLA class I concordant TG donors.** TG-derived T-cell lines (TG-TCL) from the indicated TG donors were incubated with the relevant HLA class I allele matched B-cell lines pulsed with the indicated peptides and assayed by flow cytometry for intra-cellular IFN-γ expression. The values represent the mean net percentages of live/CD3-gated IFN-γ (i.e., peptide minus mock pulsed BLCL used as antigen presenting cells) of at least 2 separate experiments.
(DOC)

Table S2 **Characteristics of HSV-1 proteins recognized by human TG-derived CD4 and CD8 T-cells.** The expression kinetics classification of the HSV-1 proteins recognized by human TG-derived CD4 and CD8 T-cells are designated as α (immediate early), β (early), γ1 (late) and γ2 (late late). Furthermore, the classification of HSV-1 proteins that are essential (E) or non-essential (nonE) for virus growth in cell culture are provided.
(DOC)
