# Supplementary figures and images for: Local CD4 and CD8 T-Cell Reactivity to HSV-1 Antigens Documents Broad Viral Protein Expression and Immune Competence in Latently Infected Human Trigeminal Ganglia
Source: PLoS Pathog. 2013 Aug 15;9(8):e1003547. doi: 10.1371/journal.ppat.1003547 (PMC3744444; doi:10.1371/journal.ppat.1003547)

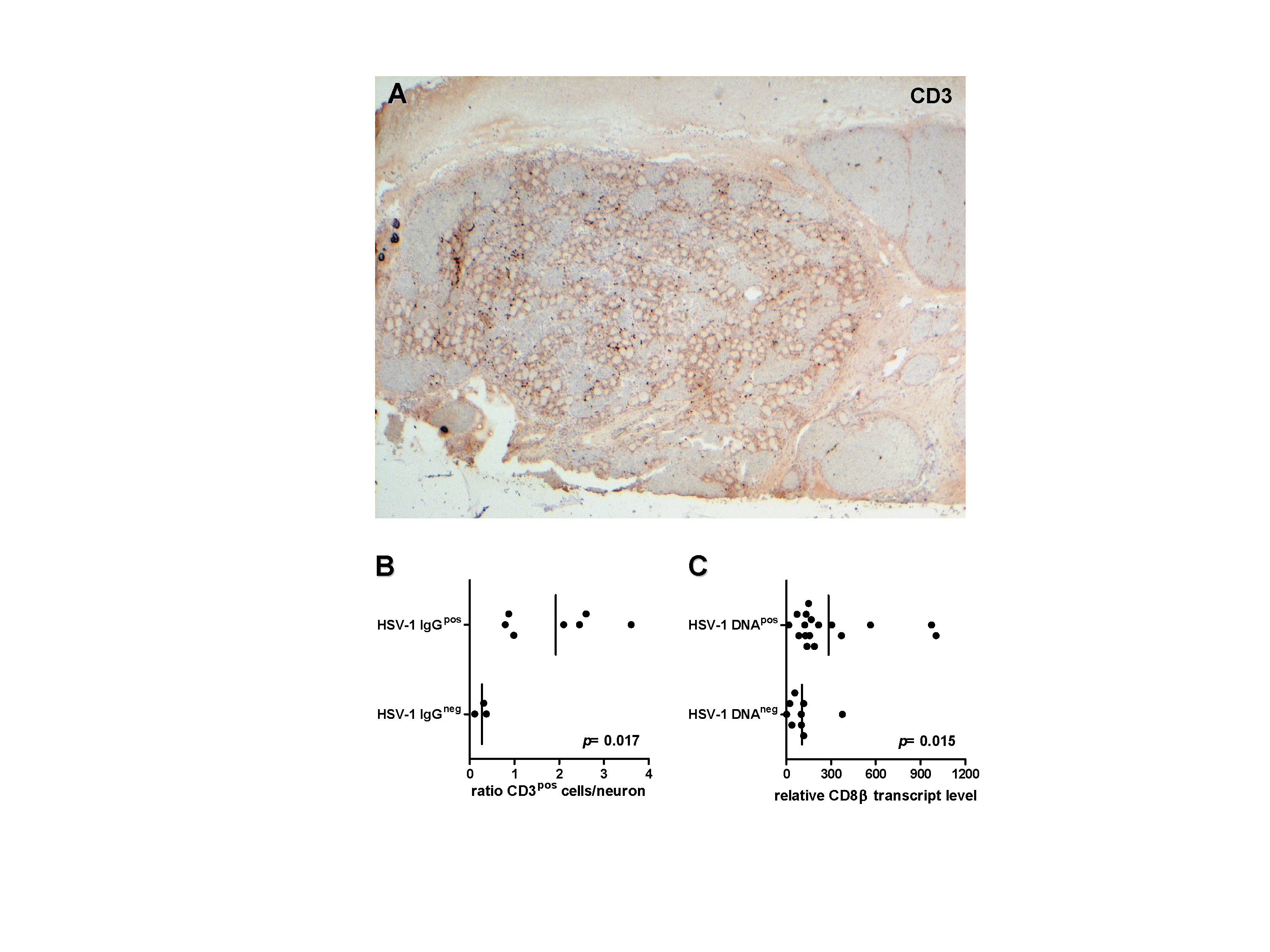

Supplement: Figure S1 — T-cell retention in human TG correlates with HSV-1 latency. (A) Representative image of a HSV-1 negative human TG stained by immunohisto-chemistry for CD3 (red). Sections were developed with 3-amino-9-ethylcarbazole (red staining pattern) and counterstained with hematoxylin (blue nuclei). Magnification was ×20. Note the limited number of infiltrating CD3+ cells that, in contrast to a TG of a representative HSV-1 positive donor (see Fig. 2A), did not form neuron-interacting T-cell clusters. Representative image from 5 HSV-1 negative TG donors analyzed. (B) Scatter plot showing the mean number of CD3+ cells per neuronal cell body in TG of HSV-1 seropositive (n = 7) and seronegative (n = 3) individuals. (C) Scatter plot showing the mean relative CD8β transcript levels in human TG that are HSV-1 DNA positive (n = 17) or negative (n = 9). (B and C) The Mann-Whitney test was used for statistical analysis. (TIFF) [file ppat.1003547.s001.tif]

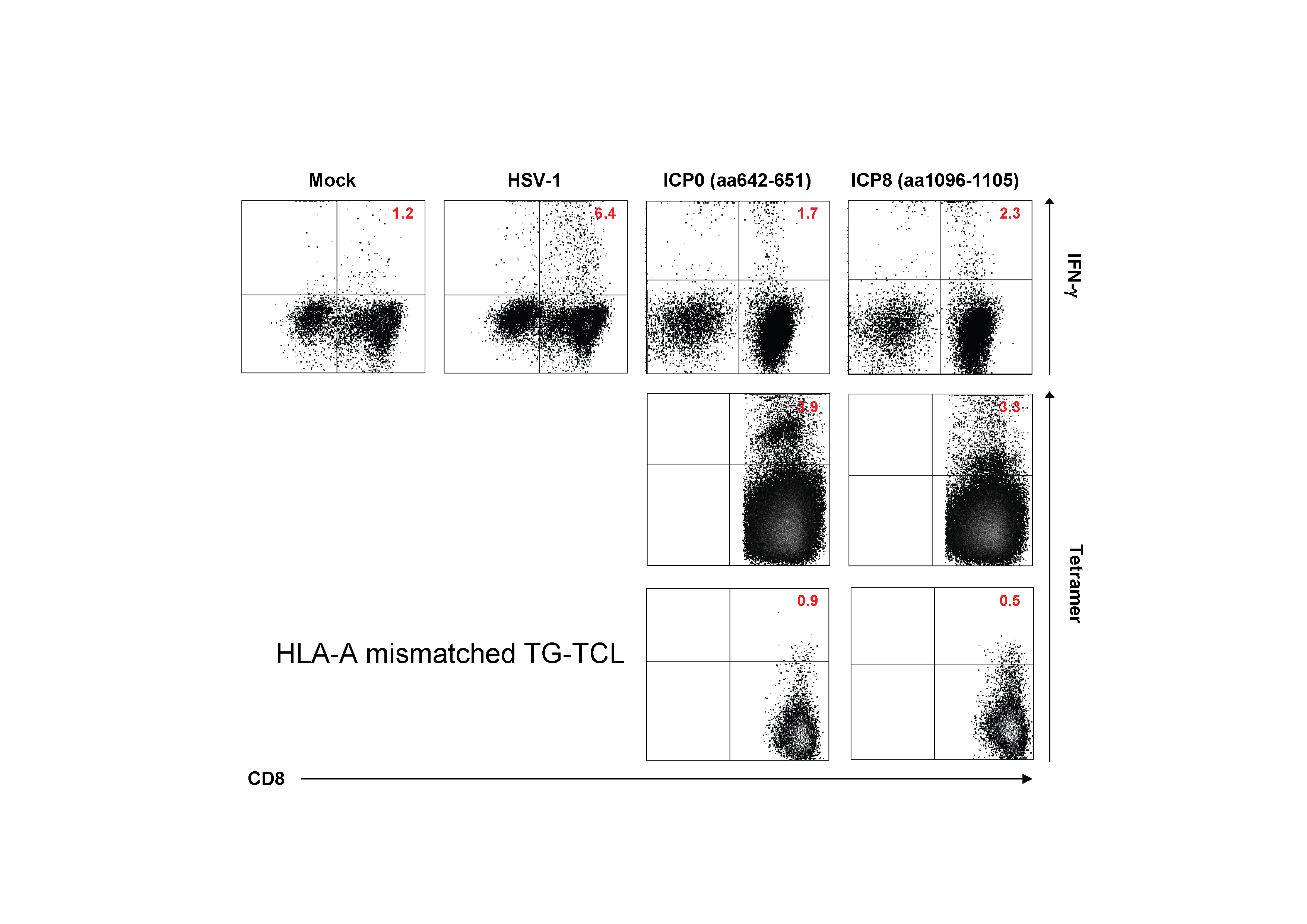

Supplement: Figure S2 — Validation of HSV-1 peptide-specific tetramers on the TG-TCL of donor TG2. The TG-TCL of donor TG2 were incubated with mock- and -HSV-1 infected, and peptide-pulsed, autologous B-cell lines for 16 hrs. Gated live and CD3+ cells were assayed by flow cytometry for intra-cellular gamma interferon (IFN-γ), and surface CD3 and CD8 expression. Additionally, the donor's TG-TCL, or a relevant HLA-A allele mismatched TG-TCL, were incubated with the corresponding fluorochrome-conjugated HLA class I tetramers for 1 hr and binding determined on live gated cells in combination with CD3 and CD8 staining (lower row). Numbers are percentages of cells in the upper right quadrant. aa, amino acid; ICP, infected cell polypeptide. (TIF) [file ppat.1003547.s002.tif]

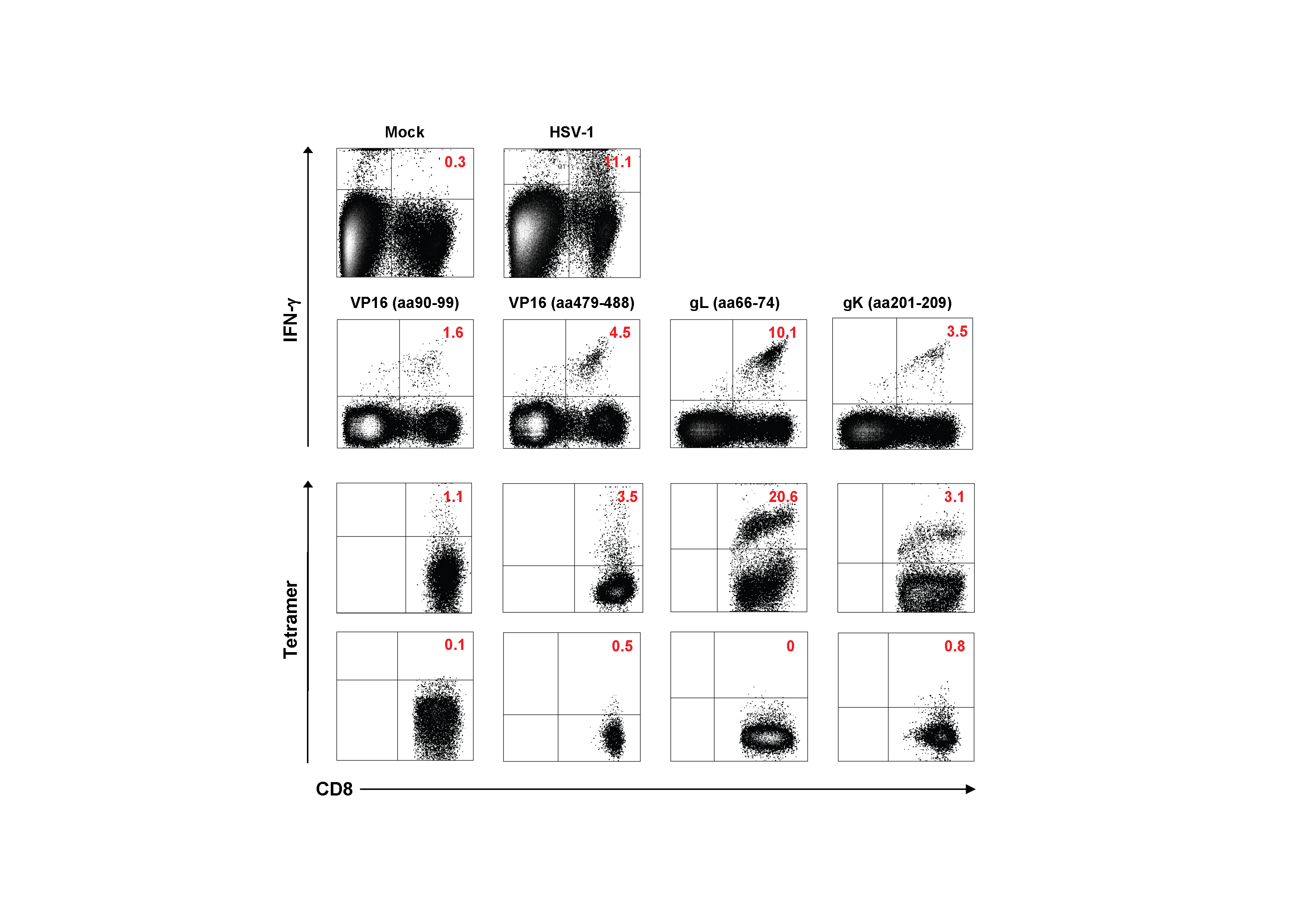

Supplement: Figure S3 — Validation of HSV-1 peptide-specific tetramers on the TG-TCL of donor TG3. The TG-TCL of donor TG3 were incubated with mock- and -HSV-1 infected, and peptide-pulsed, autologous B-cell lines for 16 hrs. Gated live and CD3+ cells assayed by flow cytometry for intra-cellular gamma interferon (IFN-γ), and surface CD3 and CD8 expression. Additionally, the donor's TG-TCL, or a HLA-A allele mismatched TG-TCL, were incubated with the corresponding fluorochrome-conjugated HLA class I tetramers for 1 hr and binding determined on live gated cells in combination with CD3 and CD8 staining (lower row). Numbers are percentages of cells in the upper right quadrant. aa, amino acid; VP, virus protein; gK, glycoprotein K and gL, glycoprotein L. (TIFF) [file ppat.1003547.s003.tif]

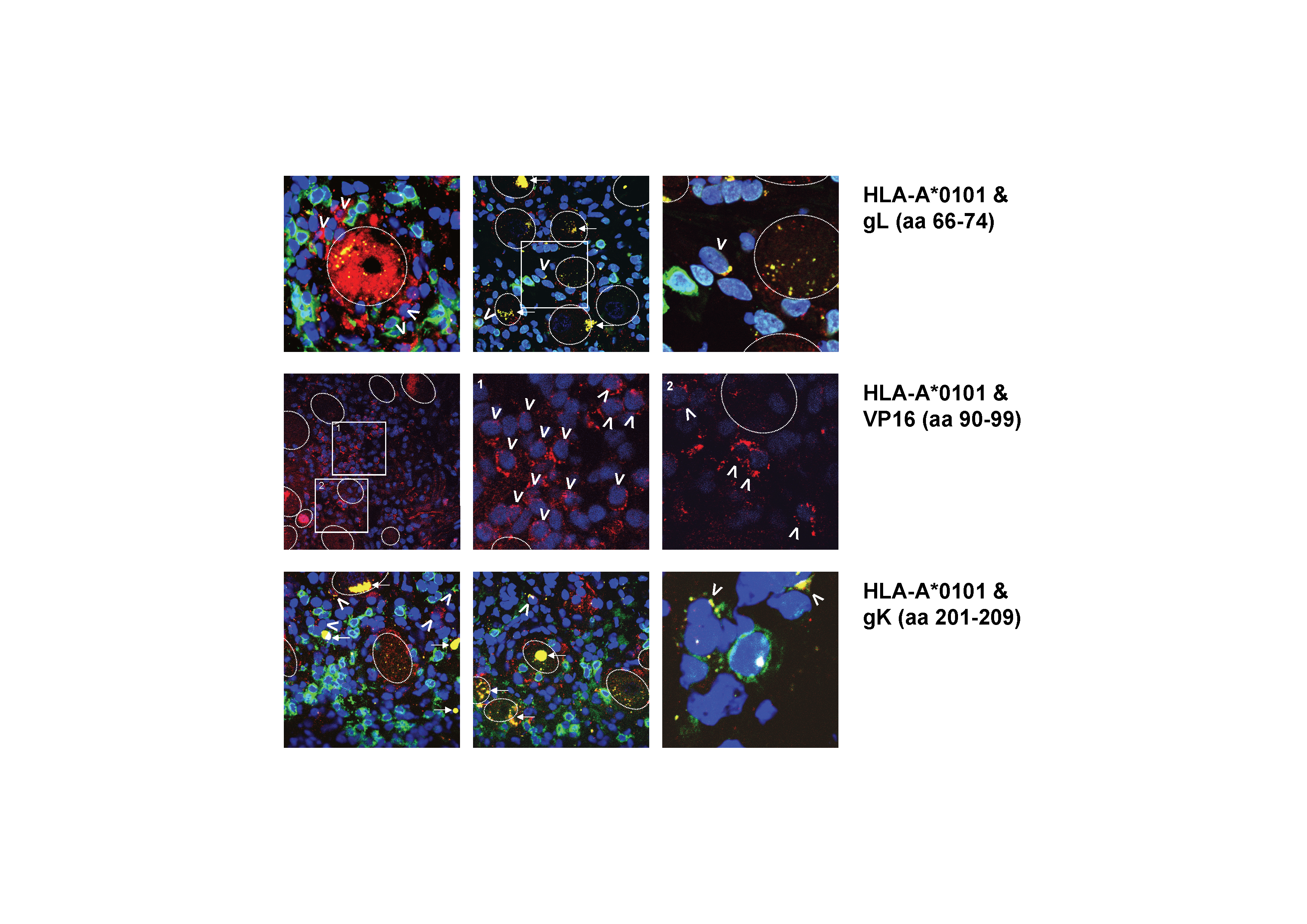

Supplement: Figure S4 — HSV-1 epitope-specific CD8 T-cells are localized in the vicinity to sensory neuron cell bodies in human TG tissue. Representative optical sections from snap-frozen TG tissue of donor TG3 stained with DAPI (blue), anti-CD8 (green) and tetramers (red) that consisted of the synthetic HSV-1 peptides gL66–74 (upper panel), VP1690–99 (middle panel) and gK201–209 (lower panel) bound to HLA-A*0101. The white arrows and arrow heads signify autofluorescent granules containing lipofuscin and tetramer-positive cells, respectively. Boxed areas in the upper and middle panels are enlarged in the corresponding images to the right. Note that for the HLA-A*0201/VP1690–99 tetramer staining anti-CD8 was omitted. Neuron outlines are marked with a white dashed line. Magnifications were ×400 and in the insets ×800. (TIFF) [file ppat.1003547.s004.tif]
