# Supplementary material for: Local CD4 and CD8 T-Cell Reactivity to HSV-1 Antigens Documents Broad Viral Protein Expression and Immune Competence in Latently Infected Human Trigeminal Ganglia
Source: PLoS Pathog. 2013 Aug 15;9(8):e1003547. doi: 10.1371/journal.ppat.1003547 (PMC3744444; doi:10.1371/journal.ppat.1003547)
Supplement: Table S1 — HSV-1 peptide responses in TG-TCL of HLA class I concordant TG donors. TG-derived T-cell lines (TG-TCL) from the indicated TG donors were incubated with the relevant HLA class I allele matched B-cell lines pulsed with the indicated peptides and assayed by flow cytometry for intra-cellular IFN-γ expression. The values represent the mean net percentages of live/CD3-gated IFN-γ (i.e., peptide minus mock pulsed BLCL used as antigen presenting cells) of at least 2 separate experiments. (DOC) [file ppat.1003547.s006.doc]

**Table S1.** HSV-1 peptide responses in TG-derived TCL of HLA class I concordant TG donors

| **HSV-1 peptide ID^*^** | | | **Percentage peptide reactive CD8 T-cells in donors’ TG-TCL^#^** | | | | | | |
| --- | --- | --- | --- | --- | --- | --- | --- | --- | --- |
| **HLA allele** | **ORF** | **aa location** | **TG1** | **TG2** | **TG3** | **TG4** | **TG6** | **TG8** | **TG9** |
| A*0101 | UL1 | 066-074 | na^d^ | na | **10** | na | na | 0 | na |
| A*0101 | UL39 | 512-520 | na | na | nd | na | na | 0 | na |
| A*0101 | UL41 | 259-268 | na | na | nd | na | na | 0 | na |
| A*0101 | UL46 | 354-362 | na | na | nd | na | na | 0 | na |
| A*0101 | UL47 | 360-368 | na | na | nd | na | na | 0 | na |
| A*0101 | UL47 | 566-574 | na | na | nd | na | na | 0 | na |
| A*0101 | UL48 | 090-099 | na | na | **1.5** | na | na | 0 | na |
| A*0101 | UL48 | 479-488 | na | na | **4** | na | na | 0 | na |
| A*0101 | UL53 | 201-209 | na | na | **3.5** | na | na | 0 | na |
|  |  |  |  |  |  |  |  |  |  |
| A*0201 | UL13 | 389-397 | 0 | 0 | na | 0 | na | 0 | 0 |
| A*0201 | UL25 | 367-375 | 0 | 0 | na | 0 | na | 0 | 0 |
| A*0201 | UL27 | 280-288 | 0 | 0 | na | 0 | na | 0 | 0 |
| A*0201 | UL27 | 448-456 | 0 | 0 | na | 0 | na | 0 | 0 |
| A*0201 | UL39 | 425-433 | 0 | 0 | na | 0 | na | 0 | 0 |
| A*0201 | UL40 | 184-192 | 0 | 0 | na | 0 | na | 0 | 0 |
| A*0201 | UL47 | 286-294 | 0 | 0 | na | 0 | na | 0 | 0 |
| A*0201 | UL47 | 374-382 | 0 | 0 | na | 0 | na | 0 | 0 |
| A*0201 | UL47 | 545-553 | 0 | 0 | na | 0 | na | 0 | 0 |
|  |  |  |  |  |  |  |  |  |  |
| A*2902 | UL25 | 170-179 | na | na | na | na | 0 | na | na |
| A*2902 | UL25 | 235-243 | na | na | na | na | 0 | na | na |
| A*2902 | UL26 | 022-030 | na | na | na | na | 0 | na | na |
| A*2902 | UL26 | 326-334 | na | na | na | na | nd | na | na |
| A*2902 | UL27 | 295-303 | na | na | na | na | 0 | na | na |
| A*2902 | UL27 | 641-649 | na | na | na | na | nd | na | na |
| A*2902 | UL29 | 460-468 | na | na | na | na | 0 | na | na |
| A*2902 | UL29 | 895-903 | na | na | na | na | 0 | na | na |
| A*2902 | UL46 | 093-101 | na | na | na | na | 0 | na | na |
| A*2902 | UL46 | 126-134 | na | na | na | na | 0 | na | na |
| A*2902 | UL46 | 224-232 | na | na | na | na | 0 | na | na |
| A*2902 | UL46 | 333-341 | na | na | na | na | 0 | na | na |
| A*2902 | UL47 | 508-516 | na | na | na | na | **1.5** | na | na |
|  |  |  |  |  |  |  |  |  |  |
| B*0702 | ICP0 | 698-706 | na | na | na | na | 0 | 0 | na |
| B*0702 | UL21 | 382-390 | na | na | na | na | 0 | 0 | na |
| B*0702 | UL49 | 281-290 | na | na | na | na | 0 | 0 | na |
| B*0702 | US1 | 070-078 | na | na | na | na | 0 | 0 | na |
| B*0702 | US7 | 022-030 | na | na | na | na | nd | nd | na |
| B*0702 | US7 | 097-105 | na | na | na | na | 0 | 0 | na |
| B*0702 | US7 | 195-203 | na | na | na | na | 0 | 0 | na |
| B*0702 | US7 | 230-238 | na | na | na | na | 0 | 0 | na |

^*^ The viral gene and encoding protein, amino acid location and the human leukocyte antigen (HLA)

presenting the indicated peptide are indicated. ORF, open reading frame and aa, amino acid.

^#^ TG-derived T-cell lines (TG-TCL) from the indicated TG donors were incubated with the relevant HLA

class I allele matched B-cell lines (BLCL) pulsed with the indicated peptides and assayed by flow cytometry

for intra-cellular IFN-γ expression. The values represent the mean net percentages of live/CD3-gated IFN-γ

(i.e., peptide minus mock pulsed BLCL used as antigen presenting cells) of at least 2 separate experiments.

HSV-1 peptides scored positive are shown in bold. na, not applicable and nd, not done.
