# Supplementary material for: Local CD4 and CD8 T-Cell Reactivity to HSV-1 Antigens Documents Broad Viral Protein Expression and Immune Competence in Latently Infected Human Trigeminal Ganglia
Source: PLoS Pathog. 2013 Aug 15;9(8):e1003547. doi: 10.1371/journal.ppat.1003547 (PMC3744444; doi:10.1371/journal.ppat.1003547)
Supplement: Table S2 — Characteristics of HSV-1 proteins recognized by human TG-derived CD4 and CD8 T-cells. The expression kinetics classification of the HSV-1 proteins recognized by human TG-derived CD4 and CD8 T-cells are designated as α (immediate early), β (early), γ1 (late) and γ2 (late late). Furthermore, the classification of HSV-1 proteins that are essential (E) or non-essential (nonE) for virus growth in cell culture are provided. (DOC) [file ppat.1003547.s007.doc]

**Table S2.** Characteristics of HSV-1 proteins recognized by human TG-derived CD4 and CD8 T-cells.

| **Gene^*^** | **Protein^#^** | **Kinetics^‡^** | **Status^§^** | **Virion^¶^** | **Function** |
| --- | --- | --- | --- | --- | --- |
| RS1 | ICP4 | α | E | Yes | Repressor/transactivator |
| RL2 | ICP0 | α | nonE | Yes | Multiple functions: e.g., virus β and γ gene regulator and IFN type 1 evasion |
| UL1 | gL | γ1 | E | Yes | Complexed with glycoprotein H, virus entry |
| UL6 | Not defined | γ1 | E | Yes | Cleavage-packaging viral DNA |
| UL23 | TK | β | nonE | Yes | Neurovirulence and target of acyclovir |
| UL25 | Not defined | γ2 | E | Yes | Virus penetration and capsid assembly |
| UL27 | gB | γ1 | E | Yes | Virus entry and syncytium formation |
| UL29 | ICP8 | β | E | No | ssDNA binding |
| UL39 | ICP6 | β | nonE | No | Large subunit of ribonucleotide reductase |
| UL46 | VP11/12 | γ1 | nonE | Yes | Tegument phosphoprotein |
| UL47 | VP13/14 | γ1 | nonE | Yes | Tegument phosphoprotein, modulates VP16 activity |
| UL48 | VP16 | γ1 | E | Yes | Tegument, pre-formed transactivator |
| UL53 | gK | γ2 | E | No | Virion exocytosis and syncytium formation |

^*^ Gene and protein names from reference 1 and Genbank NC_001806. Not all gene products have separate names.

^#^ Expression kinetics classification designated as α (immediate early), β (early), γ1 (late) and γ2 (late late). Expression of γ2 requires ongoing viral DNA synthesis. TK, thymidine kinase; gB, gK and gL, glycoproteins B, K and L, respectively. Currently, no proteins names available for HSV-1 ORFs UL6 and UL25.

^§^ Essential (E) or non-essential (nonE) for virus growth in cell culture.

^¶^ Presence or absence in highly purified virions (reference 1).
